# Molecular character of a phosphatase 2C (PP2C) gene relation to stress tolerance in *Arabidopsis thaliana*

**DOI:** 10.1007/s11033-012-2350-0

**Published:** 2012-12-26

**Authors:** Jihong Zhang, Xiushan Li, Zhimin He, Xiaoying Zhao, Qiming Wang, Bo Zhou, Dashi Yu, Xinqun Huang, Dongying Tang, Xinhong Guo, Xuanming Liu

**Affiliations:** 1 State Key Laboratory of Chemo/Biosensing and Chemometrics, College of Biology, Hunan University, Changsha, 410082 China; 2 Department of Biology Engineering, Xiangtan University, Xiangtan, 411105 China

**Keywords:** Loss-of-function mutant, GFP, GUS, Stress tolerance, Water-loss assays, Seed germination

## Abstract

Protein phosphatases type 2C (PP2Cs) from group A, which includes the ABI1/HAB1 and PP2CA branches, are key negative regulators of ABA signaling. HAI-1 gene had been shown to affect both seed and vegetative responses to ABA, which is one of PP2Cs clade A in *Arabidopsis thaliana*. Transgenic plants containing pHAI-1::GUS (β-glucuronidase) displayed GUS activity existing in the vascular system of leave veins, stems and petioles. Green fluorescent protein fused HAI-1 (HAI-1-GFP) was found in the nucleus through transient transformation assays with onion epidermal cells. The water-loss assays indicated the loss-of-function mutants did not show symptoms of wilting and they had still turgid green rosette leaves. The assays of seed germination by exogenous ABA and NaCl manifested that the loss-of-function mutants displayed higher insensitivity than wild-type plants. Taken together, the final results suggest that the HAI-1 (AT5G59220) encoded a nuclear protein and it can be highly induced by ABA and wound in *Arabidposis*, the stress-tolerance phenotype showed a slightly improvement when HAI-1 gene was disrupted.

## Introduction

Abiotic stresses adversely affect growth and productivity and trigger a series of morphological, physiological, biochemical and molecular changes in plants. ABA regulates diverse plant processes, ranging from adaptation to water stress to seed germination and dormancy [10, 18], and post-embryonic development, such as lateral root development [5, 9]. Proteins encoded by these genes, which are ABA-mediated processes are important for plant tolerance to several abiotic stresses including salt, drought and freezing [30, 43].

Although numerous factors related to ABA responses had been reported [19], however, the ABA signaling model was dramatically updated due to two findings. Recently, a core signaling pathway has been established by the discovery of PYR/PYL/RCAR as a new type of soluble ABA receptor [33, 40], and the identification of a protein phosphatase–kinase complex [type 2C protein phosphatase (PP2C)-SNF1-related protein kinase 2 (SnRK2)] as downstream components of PYR/PYL/RCARs [49]. SRK2E plays a key role in stomatal responses to ABA in *Arabidopsis* [52, 55], while SRK2D and SRK21 are probably involved in ABA signaling during seed germination and root growth [12].

Salt stress, drought and, to a lesser extent, cold stress elevate ABA levels [35]. ABA upregulates the expression of many, but not all, drought-response genes [25, 31], indicating that there are ABA-dependent and ABA-independent salt and drought stress responses in plants [54].

Sixty-nine PP2Cs are encoded in the *Arabidopsis* genome [22, 47], according to sequence alignment of the catalytic phosphatase core, the clade A of PP2Cs is arranged in two subgroups, one including ABI1, ABI2, HAB1 and HAB2, and a second one formed by PP2CA/AHG3, AHG1, At5g59220, At1g07430 and At2g29380 [47], have largely overlapping but different roles as negative regulators of ABA signaling, mainly during germination [19]. The recessive loss-of-function mutants *hab1*-*1* shows ABA hypersensitive inhibition of seed germination and enhanced ABA-mediated stomatal closure [26]. Genetic evidence has largely supported the negative role of PP2Cs in ABA signaling, and certain triple loss-of-function pp2c mutants display partial constitutive response to ABA [44].

Among the *ABI* genes, *ABI1* and *ABI2* are unique in that they encode PP2Cs, which are ubiquitously found in all eukaryotes and involved in phosphorylation-mediated signaling; In addition, these genes function through seed maturation and germination to vegetative growth. The mutants show a broad range of ABA-related phenotypes, including reduced seed dormancy, ABA-resistant seed germination and seedling growth, abnormal stomatal regulation, and defects in various responses to drought [11, 24].

Both PP2CA/AHG3 and AHG1 appear to play an essential role for ABA signaling during seed development and germination [26, 36, 57], but in contrast to *pp2ca*-*1*, the *ahg1*-*1* mutant has no ABA-related phenotype in adult plants and expression of AHG1 is restricted to seed [36].

HAB1 is broadly expressed in the plant and strongly induced by ABA [29, 45]. Constitutive expression of HAB1 under a 35S promoter led to reduced ABA sensitivity both in seeds and vegetative tissues, compared to wild-type plants [45].

We previously reported that group A PP2Cs is functionally redundant at the molecular level, but they have distinctive roles in different tissues and organs, as indicated by tissue-specific expression patterns. The plant PP2CA genes appear to be expressed ubiquitously in various organs, albeit at varying levels. ABI1 is expressed in various tissues, including seeds and guard cells, and AHG1 and AHG3 are specifically localized in the nucleus [49]. Therefore, we took advantage of transgenic plants containing pHAI-1::GUS (β-glucuronidase) to study the expression of HAI-1 gene. To test the role of HAI-1 gene in *Arabidopsis*, we had constructed over-expression transgenic plants and gained the homozygote mutants of *hai*-*1*, which the T-DNA insertion was in the HAI-1 gene.

In this study, we successfully isolated from the T-DNA insertion mutant of HAI-1, namely *hai*-*1*. Furthermore, the mutants of *hai*-*1* showed greatly enhanced tolerance to drought stress and highly enhanced ABA or NaCl insensitivity. Moreover, HAI-1 gene (highly ABA-induced PP2C gene 1) was highly induced by wound and ABA through generating transgenic *Arabidopsis* plants, which were carrying the HAI-1 promoters fused to the GUS gene.

## Materials and methods

### Plant material and growth conditions

*Arabidopsis* *thaliana* L. Heynh. Ecotype columbia was used in this study unless otherwise indicated. Plant growth conditions have been described elsewhere [37].

### Transgenic plants

Using genomic sequences from the TAIR database (http://www.arabidopsis.org), the full-length HAI1 (AT5G59220) open reading frame were amplified from cDNA by PCR with primers Pro-F (sense, 5′-GGGGACAAGTTTGTACAAAAAAGCAGGCTTCTGAATATCTTATAATTTTTGCCC-3′) and Pro-R (antisense, 5′-GGGGACCACTTTGTACAAGAAAGCTGGGTGTCTCTTCTCCTCCGCCTCTGTAA-3′) and were inserted into pDONR201 Entry vector (Promega) by Gateway cloning technology and sequenced then was inserted into 35S pleela vector by LB clonase (Invitrogen company). *Agrobacterium* GV3101 90RK were transformed with these plasmids and used for infection of flowering plants by the floral dip method [7].

### Loss-of-function insertion lines

Loss-of-function lines were obtained from the *Arabidopsis* biological resource center (ABRC). 7-day-old homozygous plants were identified by the kanamycin tolerance test and a PCR-based method using loss-of-function left- or right- border primers [1].

### Root growth and germination assays

The root growth assay for scoring ABA sensitivity was performed by measuring root growth after 3 days cold treatment and 6-day-old seedlings were growing onto MS plates. To measure ABA sensitivity, seeds were plated on solid medium, composed of MS basal salts, 3 % sucrose and increasing concentrations of ABA (0, 0.1 0.3, 0.6 or 1 μM) and of NaCl (0, 20, 50 or 70 mM) [22]. In order to score seed germination, the percentage of seeds which had germinated and developed fully green expanded cotyledons was determined.

### Drought stress and water-loss assays

The two different water-loss assays were performed. The short-term water-loss assays were performed in detached leaves at the same developmental stage and size from 20-day-old plants. The short-term water-term assay was been described previously [2]. Four samples of five leaves per genotype were excised and fresh weight was determined by submitting the leaves to the drying atmosphere of greenhouse at 22 °C for 4 h. Data were averages ± SE from three independent experiments (*n* = 5). The difference in weight was considered as water loss.

Long-term water-loss assays were performed after removing watering in 20-day-old plants maintained under greenhouse conditions. To this end, plants (10 individuals per experiment, three independent experiments) were grown under normal watering conditions for 21 days and then subjected to drought stress by completely terminating irrigation under greenhouse conditions [44].

### Construction of pHAI-1::GUS and histochemical staining

The promoter region was amplified from the genomic DNA of Columbia ecotype and fused to a GUS reporter gene in the binary vector pGKB5, which contained a promoterless GUS reporter gene fused to the right border, and the genes conferring kanamycin and Basta resistance as plant selection markers with primers as Table 1 using the gateway cloning system (invitrogen) [51]. The binary plasmid derived from a pBGS plasmid [48], harbors a bacterial kanamycin resistance gene, and is able to replicate both in *Escherichia coli* and in Agrobacterium. The GUS-nos3′ reporter cassette from pBI101.1 [21]. The constructed fusion expression vector pGKB5 was introduced into Columbia by vacuum infiltration, and T_2_ lines were used for analysis. GUS staining was performed for 2–3 h at 37 °C using X-Gluc (Sigma-Aldrich, St. Louis, MO, USA) as described previously [21]. Finally, the tissue was mounted in 70 % ethanol on a glass microscope slide and observed under an olympus BX51 microscope (Olympus Corporation, Japan), as described previously [50]. Histochemical GUS reporter assay was performed.

**Table 1:** Primers used for PCR and qPCR analysis

Genes	Forward (5′–3′)	Reverse (5′–3′)
PP2CA2oxF	GGGGACAAGTTTGTACAAAAAAGCAGGCTTCCCGGAATTCATGGCTGAGATTTGTTACGAGAA	GGGGACCACTTTGTACAAGAAAGCTGGGTGCCGCCCGGGTTACTTATGATCGGAGGATAAAGCAA
PP2CA2oxB	GGGGACAAGTTTGTACAAAAAAGCAGGCTTCCCGGAATTCATGCCAGACCGTC CGGACGA	GGGGACCACTTTGTACAAGAAAGCTGGGTGCCGCCCGGGCTACGTGTCTCGTCGTAGATCA
pOK	TGGTTCACGTAGTGGGCCATCG	
pLB	AGAAGTATTCACGCACCAAGG	AATCTCAGCCATGTGATCGTC
59220Promoter	GGGGACAAGTTTGTACAAAAAAGCAGGCTTCTGAATATCTTATAATTTTTGCCC	GGGGACCACTTTGTACAAGAAAGCTGGGTGTCTCTTCTCCTCCGCCTCTGTAA
PP2CA2ox	ATGGCTGAGATTTGTTACG	CTACGTGTCTCGTCGTAGA
59220pEDST17	GGGGACAAGTTTGTACAAAAAAGCAGGCTTC ATGGCTGAGATTTGTTACG	GGGGACCACTTTGTACAAGAAAGCTGGGTG CTACGTGTCTCGTCGTAGA
Actin2	CACTGTGCCAATCTACGAGGGT	CACAAACGAGGGCTGGAACAAG

### Bombardment experiments

Each plate was shot twice. Each shot contains 270 μg gold particles (1.0 μm in diameter), and particles were coated with 2 μl of p35S:HAI-1-GFP recombinant plasmid at 0.5 μg/μl. The gold-coated DNA particles were delivered into onion epidermal cells using the PDS-1000/He biolistic particle delivery system (BioRad Laboratories, Hercules, CA, USA), and the bombarded onion epidermal peels were maintained at 25 °C for at least 12 h until they were examined by fluorescence microscopy (Nikon, Tokyo, Japan) [51].

## Results

### Physiological characterization of *hai*-*1* mutants

**Fig. 1:**
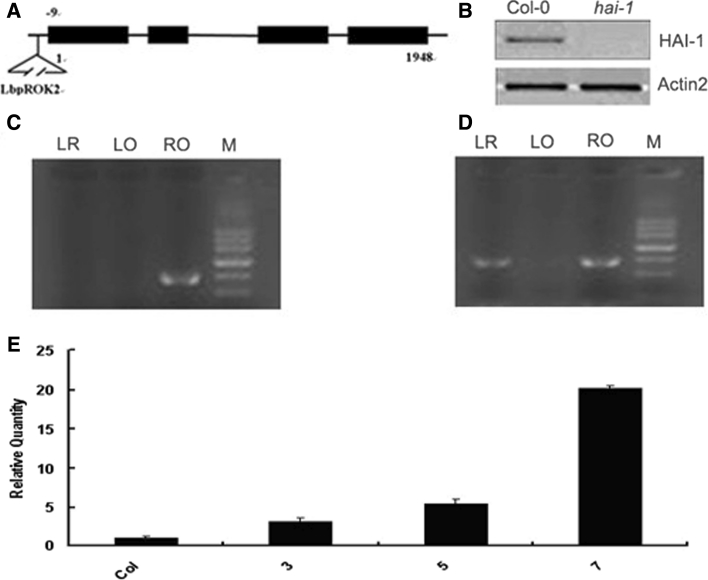
Physiological characterization of loss-of-function mutant *hai*-*1* and HAI-1 over-expression transgenic plants **a** Diagrammatic representation of the T-DNA insertions in the At5g59220 gene. **b** RT-PCR analysis of mRNAs from wt and *hai*-*1* mutant seedlings. **c** The loss-of-function mutant of *HAI*-*1* gene was identified as homozygous mutant by gel electrophoresis. **d** The loss-of-function mutant of *HAI*-*1* gene was identified as heterozygous mutant by gel electrophoresis. **e** Relative expression levels of *HAI*-*1* gene in HAI-1 over-expression transgenic plants (3, 5 and 7) compared to wild type by Q-PCR analysis. The expression level of *HAI*-*1* gene in Col-0 was defined as “1”

T-DNA insertion mutants of HAI-1 were identified in the Salk collection (Columbia background), corresponding to donor stock number Salk_039723, and it was named *hai*-*1*. Sequencing of the T-DNA flanking region in *hai*-*1* showed that the insertion was localized nine nucleotides upstream of the ATG start codon (Fig. 1a). T-DNA insertions severely impaired HAI-1 expression, based on reverse transcription (RT) -PCR (Fig. 1b). Homozygous individuals were identified by three primers PCR method. The primers of sequences of T-DNA insertion loci of flanking region both sides and within T-DNA insertion fragment were designed, homozygous loss-of-function mutants were identified from genomic DNA with pOK/pLB/pRB primers (Table 1). PCR amplification results were shown that there was an electrophoretic band amplified from homozygous genomic DNA. Heterozygous genomic DNA can amplify two electrophoretic bands (Fig. 1c, d).

To further elucidate the role of *HAI*-*1* in ABA signaling, we designed the primers according to its cDNA sequence of *HAI*-*1* gene from 1 to 1,242 bp by high fidelity PCR polymerase. Making use of gateway system BP and LR reaction, the sequences were cloned into the entry vector pDONR201 and the expression vector pLeela, respectively. Homozygous mutant plants were identified by Q-PCR. The *HAI*-*1* gene expression levels of over-expression transgenic lines 3, 5 and 7 were higher than that of Col-0 (Fig. 1e).

### Phenotypic characteristics of over-expression mutants and wild type

**Fig. 2:**
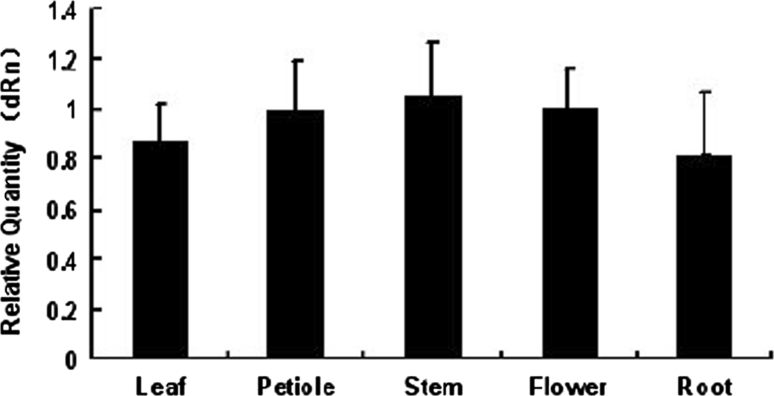
The expression of *HAI*-*1* gene in different tissues, including stem, root, leaf, petiole and flower of 15-day-old Col-0 seedlings by Q-PCR analyses

**Fig. 3:**
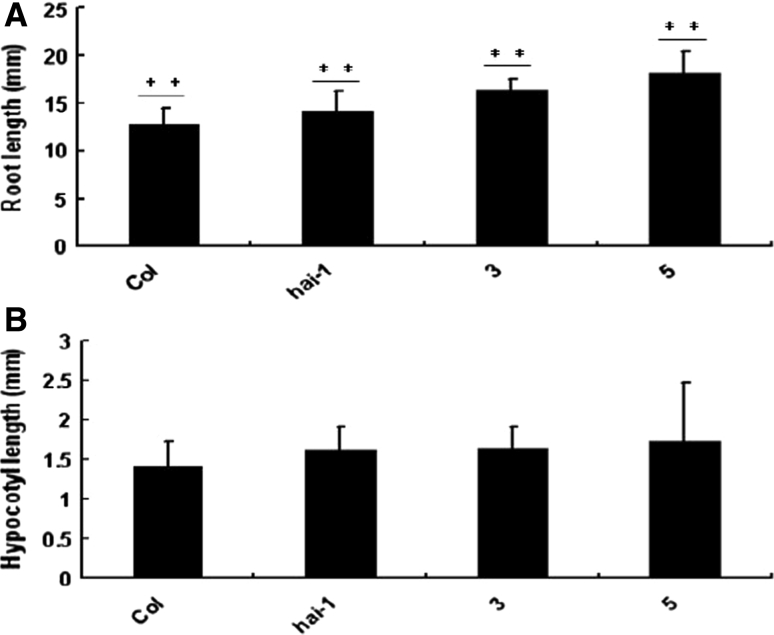
Phenotypes analysis of mutant plants **a** The means of hypocotyl lengths of seedlings of wild type, loss-of-function mutants and HAI-1 over-expression transgenic plants are shown. **b** The means of root lengths of seedlings of wild type, loss-of-function mutant and HAI-1 over-expression transgenic plants are shown. 6-day-old seedlings were scored after cold treatment. Values are averages ± SD (*n* = 30). *P ≤ 0.05, **P ≤ 0.01

To examine the expression patterns in various young tissues, total RNA were extracted from different organs of 7-day-old Col-0 wild type plants. Q-PCR analyses showed that the higher levels of HAI-1 mRNA were observed in stems and flowers, and expressed at slightly lower levels in all of the organs (Fig. 2). 6-day-old seedlings of Col-0, loss-of-function mutants and HAI-1 over-expression transgenic plants were exposed to natural condition. There were a few differences in root length and hypocotyl length among these mutants. The root length and hypocotyl length of over-expression mutants were longer than those of loss-of-function mutants and Col-0 (Fig. 3).

### Mutants of *hai*-*1* confer NaCl insensitivity

**Fig. 4:**
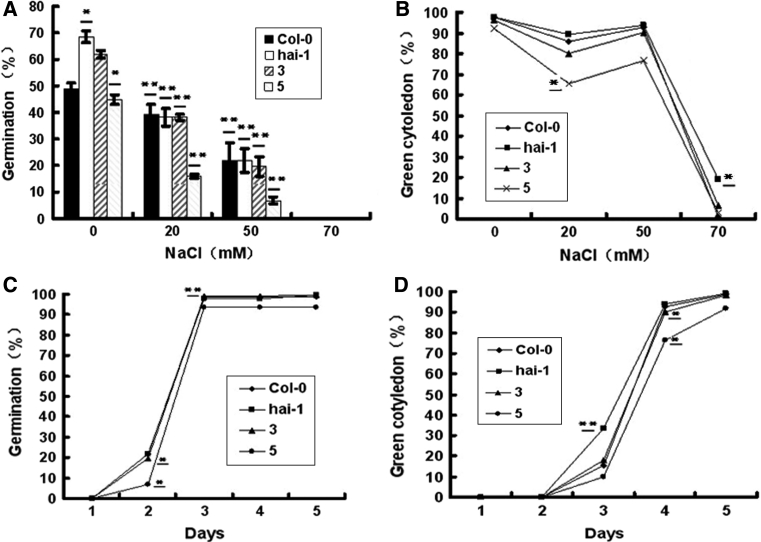
NaCl-insensitive germination inhibition of *hai*-*1* mutants as compared to wild-type and over-expression transgenic plant seeds **a** *hai*-*1* mutants showed more NaCl-insensitive seed germination response at 2 days after cold treatment, **b** *hai*-*1* mutants showed more green cotyledon percentage in the presence of various NaCl concentrations at 4 days after cold treatment, **c** seed germination kinetic profiles were determined in the presence of 50 mM NaCl, **d** green cotyledon percentage kinetic profiles were determined in the presence of 50 mM NaCl. Values are averages ± SD (*n* = 40–50), three replicates. *P ≤ 0.05, **P ≤ 0.01

We then subjected the mutant seeds in the presence of various concentrations of NaCl (0, 20, 50 or 70 mM). A dose–response curve showed germination rates of mutant plants clearly decreased with increasing NaCl concentration at 2 days after stratification (Fig. 4a). The green cotyledon percentage of *hai*-*1* is higher than that of others at 4 days after stratification in the presence of 20 mM NaCl. At 50 mM, the green cotyledon percentage of *hai*-*1* mutants appear almost identical to that of wild-type plants (Fig. 4b).

**Fig. 5:**
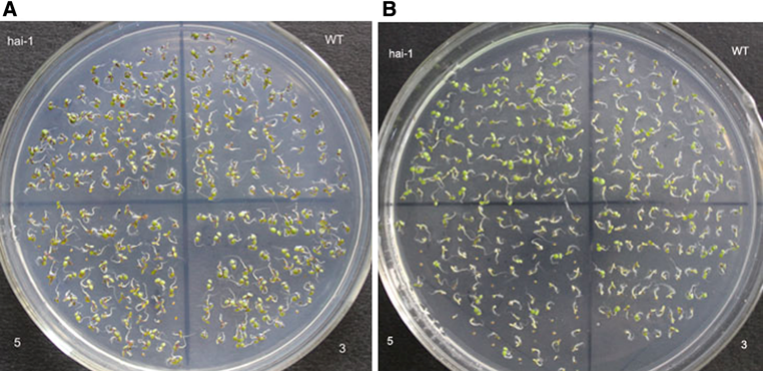
*hai*-*1* mutants enhance NaCl-insensitive inhibition germination **a** The seed germination was determined in the presence of 20 mM NaCl. **b** The seed germination was determined in the presence of 70 mM NaCl. Photographs were taken at 5 days after cold treatment; 3, 5 represented HAI-1 over-expression transgenic plant lines

The germination and green cotyledon kinetic profile showed *hai*-*1* mutants had higher germination rate and green cotyledon percentage than the wild type and over-expression transgenic plants; especially over-expression line 5 was seen clearly significant (Fig. 4c, d). We can intuitively know about the germination rates and green cotyledon percentages were almost the same with 20 mM NaCl (Fig. 5a). However, the green cotyledon percentage of *hai*-*1* mutants was higher than those of wild type and over-expression transgenic plants with 70 mM NaCl at 5 days (Fig. 5b). These experiments indicate that *HAI*-*1* gene probably has sensitive function in NaCl-mediated seed germination inhibition response.

### Mutants of *hai*-*1* confer ABA insensitivity

**Fig. 6:**
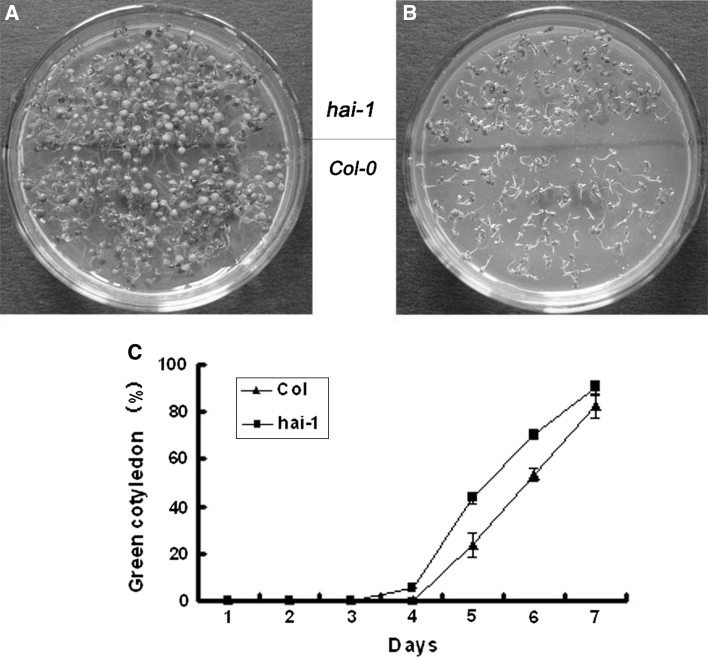
Mutants of *hai*-*1* slightly inhibit ABA response in seed germination. The comparison of seed germination sensitivity to the ABA stress between wild type and *hai*-*1* mutant plants. Photographs were taken at 6 days after cold treatment. In MS culture without ABA (**a**) or with 0.1 μM ABA (**b**), **c** *hai*-*1* mutants showed slightly higher green cotyledon percentage kinetic profiles with 0.1 μM ABA

In order to investigate the relative contribution of At5g59220 to ABA signaling, we analyzed ABA-response of the At5g59220 loss-of-function mutant Salk_039723, which we have named *hai*-*1*. After 6 days culture on the medium containing 0 or 0.1 μM ABA, wild type (Col) and the loss-of-function mutant (*hai*-*1*) had the same germination inhibition (Data no shown). But the green cotyledon percentage of *hai*-*1* was slightly higher than Col-0, the loss-of-function mutant *hai*-*1* is insensitive to ABA response than Col-0 (Fig. 6).

**Fig. 7:**
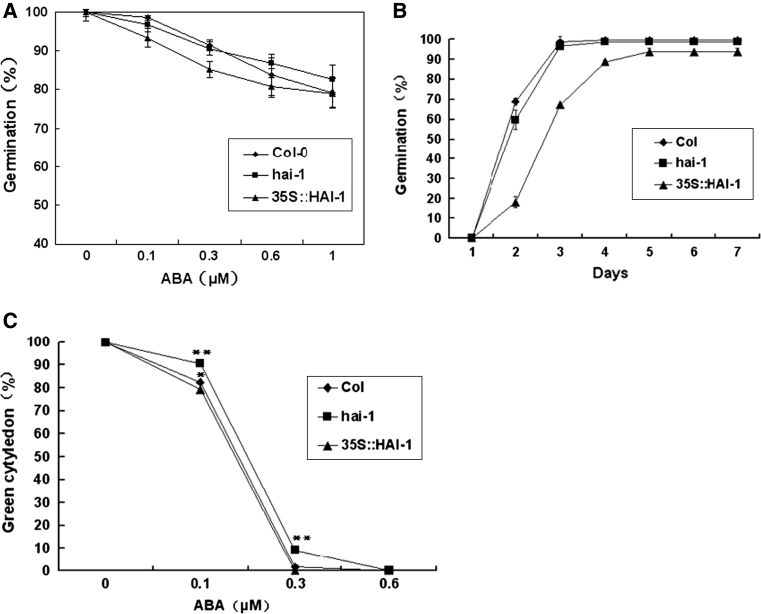
Mutants of *hai*-*1* were insensitive to ABA in seed germination inhibition. **a** Germination for ABA dose–response was scored at 3 days after cold treatment. **b** Over-expression transgenic mutants 35S::HAI-1 showed lower seed germination kinetic profiles with 0.1 μM ABA. **c** The green cotyledon percentage kinetic profiles of various mutants and wild type are shown in the presence of various ABA concentrations (0, 0.1, 0.3 or 0.6 μM) at 7 days after cold treatment. Values are averages ± SD (*n* = 40–50), three replicates. *P ≤ 0.05, **P ≤ 0.01

We then subjected the mutant seeds in the presence of various concentrations of ABA (0, 0.1, 0.3, 0.6 or 1 μM). The germination kinetic profile showed that more loss-of-function insertion mutant seeds germinated than wild-type seeds and over-expression mutant seeds at 3 days after stratification (Fig. 7a). The mutants of *hai*-*1* had higher seed germination rates than the wild type and over-expression transgenic plants for 5 days in the presence of 0.1 μM ABA (Fig. 7b), the germination rates of all mutants eventually reach to 100 % at 7 days (Fig. 7b). The more obvious reduction in germination rate for the over-expression transgenic plants than wild-type and loss-of-function mutant plants. A dose–response curve showed that after 7 days in the presence of 0, 0.1, 0.3 or 0.6 μM ABA, the green cotyledon percentage of *hai*-*1* mutants was always higher than that of over-expression transgenic plants and wild type plants (Fig. 7c).

### Adaptive responses to drought stress

Water-loss data was obtained, under greenhouse conditions, after exposing 20-day-old plants to drought stress by completely withholding water. Figure 9a showed after 12 days without watering, wild-type plants wilted and many rosette leaves yellowed. A limited improvement was observed under these conditions, *hai*-*1* mutants showed a reduced water loss as compared to wild type and over-expression transgenic plants.

**Fig. 8:**
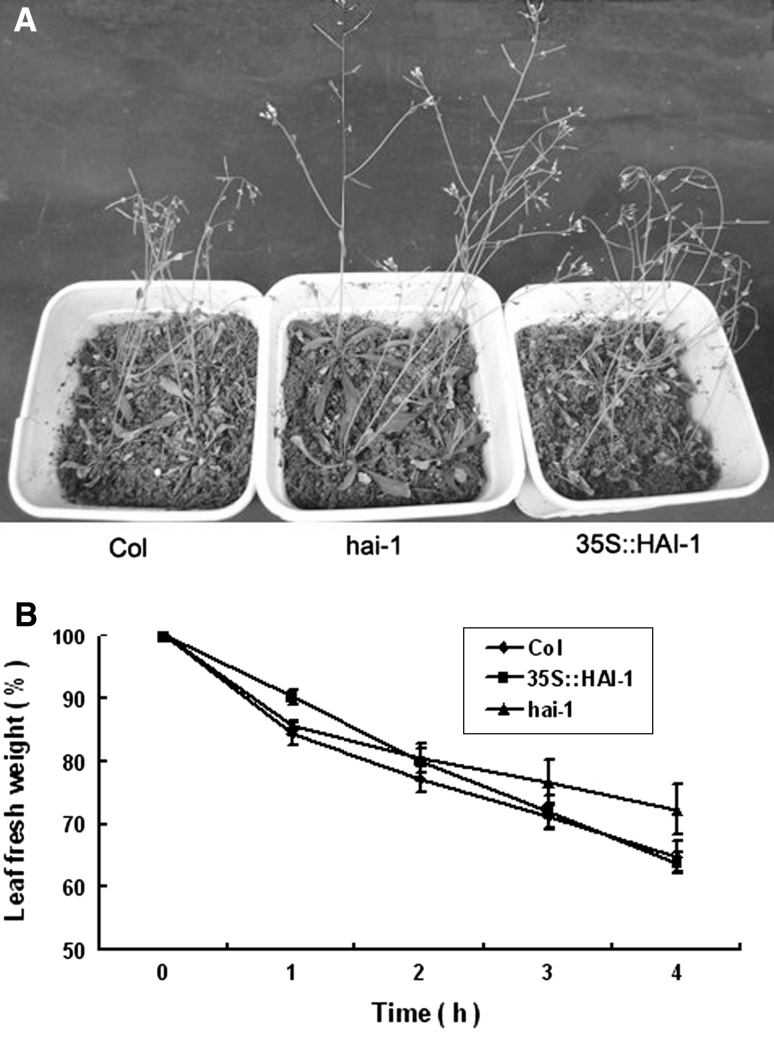
Water-loss assays was obtained **a** Enhanced drought resistance of *hai*-*1* mutant plants with respect to wild type or over-expression transgenic plants. Photographs were taken 12 days after withholding water. **b** Reduced water loss measured in detached leaves of *hai*-*1* mutants as compared to wild type or its over-expression transgenic plants. Values are averages from two independent experiments (*n* = 5)

Under these experimental conditions, The HAI-1 over-expression transgenic plants did not exhibit significant differences in the transpiration rate of detached leaves compared to wild type (Fig. 8b). However, loss-of-function mutants exhibited a reduced water loss (Fig. 8b).

Detached-leaf water-loss assays are likely not sensitive enough as to detect such variations [11], which are apparent after long periods of drought. Thus, whereas wild-type plants exhibited a marked water loss under these conditions, the ABA-hypersensitive mutants exhibited a reduced water loss, particularly in the case of over-expression transgenic plants. This result was consistent with previous studies conducted in USA [2].

### The expression of HAI-1 in pHAI1: GUS transgenic plants

**Fig. 9:**
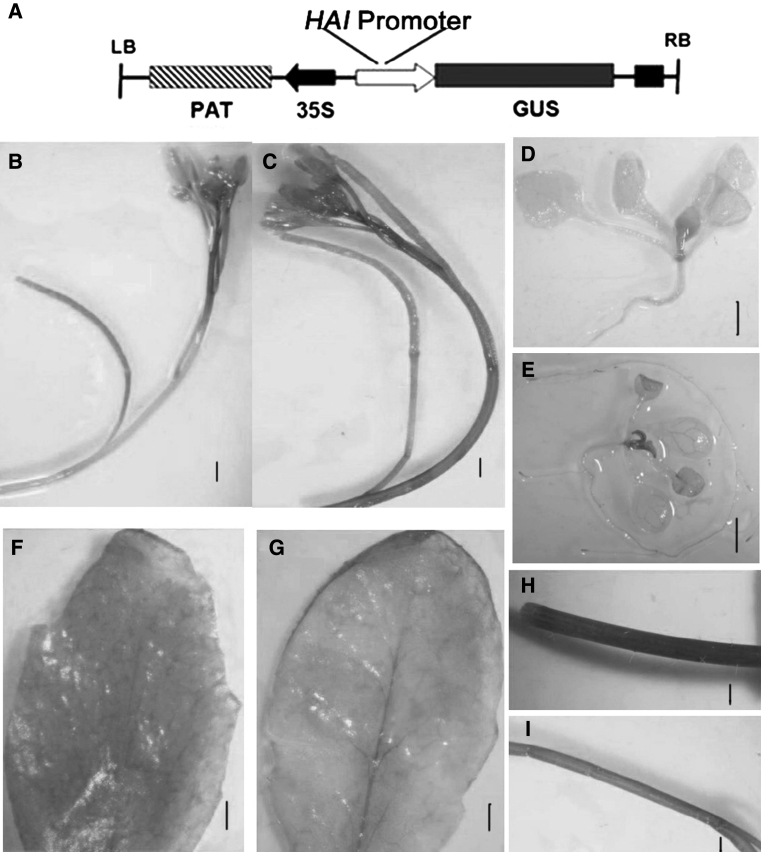
Expression patterns of HAI-1 as revealed by pHAI-1::GUS. **a** The schematic diagram of expression vector Promoter::Gw-GUS. **b** An inflorescence with flowers and stems without ABA retreatment. **c** An inflorescence with flowers and stems with 1 μM ABA retreatment for 2 h. **d** 8-day-old seedlings without ABA retreatment. **e** 8-day-old seedlings with 1 μM ABA retreatment. **f** Unwounded mature leave, **g** wounded mature leave. **h** Wounded stem cut-side. I, stem with 1 μM ABA retreatment for 2 h. **b**, **c**, **f**–**i** different type parts of a 30-day-old transgenic plant grown on soil. *Bars* 0.3 cm (**b**, **c**), 1 cm (**d**, **e**), 0.2 cm (**f**, **g**). 0.5 cm (**h**, **i**)

The 1,681 bp upstream region and 221 bp downstream region of the translational start codon ATG of HAI-1 was fused to the GUS gene, and the resulting construct was transformed into wild-type *Arabidopsis* plants to produce a set of 18 independent pHAI1:GUS lines. GUS activities were not detected in 8-day-old transgenic seedling’s leaf vein, root, or stem, and except only a little expressed in stem primordia. While the GUS staining can be detectable in whole seeding after induced 2 h by 1 μM ABA, including in root tip, in stem primordia, and in leaf vein (Fig. 9c, d). GUS activities were abundantly detected in the stem and leaf vein of 3–4 week-old mature transgenic plants, but barely detected in young silique after ABA implied (Fig. 9a, b). In addition, we also found that the HAI-1 promoter was induced under ABA and abiotic stresses such as wounding, GUS activities were largely observed at the wound around, such as in leaf vascular system and in around cut-side stem (Fig. 9e–h).

### HAI-1 protein was localized to the nucleus

**Fig. 10:**
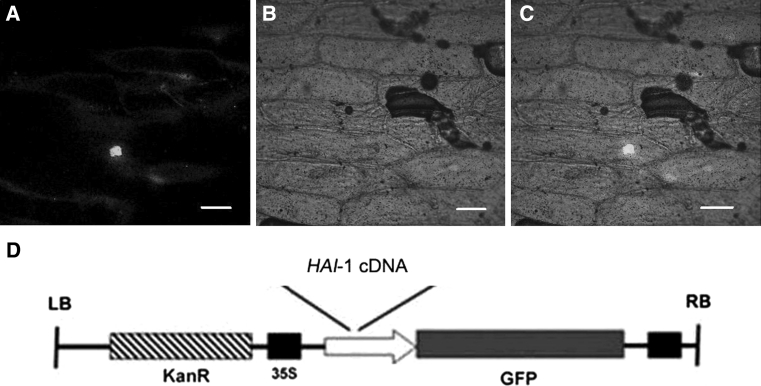
Transient expression of HAI-1-GFP in the bombarded onion epidermal peels. The HAI-1-GFP fluorescence was found to be localized to the nucleus. **a**–**c** Fluorescence image, bright field image and merged fluorescence image of 35S::HAI-1-GFP. *Bars* 100 μm. **d** The schematic diagram of HAI-1:GFP fusion vector

Proper elucidation of the subcellular localization of clade A PP2Cs is an important goal to better understand their role in plant physiology. To investigate the subcellular localization of HAI-1 protein, we fused the coding region of HAI1 to N-terminus of GFP under the control of cauliflower mosaic virus (CaMV) 35S promoter. The HAI-1-GFP fusion was both tested by bombardment assay into onion epidermal cells. Gene expression was revealed by the green fluorescence of the GFP marker and observed under a confocal microscope. The HAI-1-GFP fusion protein was localized to the nucleus in onion epidermal cells (Fig. 10).

## Conclusion and discussion

According to the results of Q-PCR, the expression level of HAI-1 was very low (Fig. 2). At5g59220 is not expressed in seeds [34, 36, 57], but it is expressed in seedlings or different tissues of adult plants according to public microarray data [3].

ABA has an inhibitory effect on root growth, consequently, ABA-insensitive mutants are resistant to this ABA-mediated process [17]. In seeds, ABA has been shown to play an important role in the formation, maintenance of dormancy, and inhibition of germination [4]. The mutants of *hai*-*1* had higher seed germination rates and green cotyledon percentages than the wild type and over-expression transgenic plants in the presence of exogenous ABA (Fig. 7). The results were amazingly consistent with the prior findings; the *abi1*-*1* mutant displays a lower sensitivity than the wild type to the inhibition of seed germination by exogenous ABA [24].

In this study, we identify and characterize At5g59220 (HAI-1) gene disruption and over-expression phenotypes in *Arabidopsis*. The loss-of-function mutants *hai*-*1* result in a strongly increased insensitivity to ABA during seed germination (Fig. 7). Moreover, constitutive expression of *HAI*-*1* gene enhanced ABA-hypersensitive to exogenously applied ABA. ABA biosynthetic and signaling pathways can be considered as potential targets to improve plant performance under drought. Thus, it has been demonstrated that transgenic plants producing high levels of ABA display improved growth under drought stress than wild type [20, 42]. Alternatively, mutants affected in ABA signal transduction might also show an enhanced ABA response leading to stress-tolerant phenotypes.

Many examples of ABA-hypersensitive mutants have been reported [57]; however, in spite of the critical role of ABA to coordinate plant response to drought, a general correlation between enhanced response to ABA and drought tolerance has not been well established. The results showed after 12 days without watering, wild-type plants wilted and many rosette leaves yellowed (Fig. 8a). While amplified segment expression mutants showed the same phenotype as loss-of-function mutants (Fig. 8a). A limited improvement was observed under these conditions the single *hai*-*1* mutants showed a reduced water loss as compared to wild type and over-expression mutants (Fig. 8).

Although some mutants with enhanced response to ABA have been shown to cause reduced water consumption [14, 38, 41], many examples of mutants that do not match this assertion is known. For instance, the mutants, which show ABA- hypersensitive inhibition of seed germination and super-induction of ABA-responsive genes, have compromised tolerance to drought stress [15, 53]. However, Mutants displayed ABA hypersensitivity and enhanced expression of ABA signaling genes did not correlate with stress-tolerance phenotypes [23, 38, 39]. The over-expression transgenic plants exhibited an enhanced water loss (Fig. 8b), the reason might be ABA- hypersensitive inhibition of seed germination had compromised tolerance to drought stress.

Previous leaf water loss analyses from detached leaves have shown that these assays can show phenotypic differences in mutations in which steady-state stomatal apertures already differ from wild-type controls prior to excising leaves [8, 27, 32]. The results showed mutants *hai*-*1* display ABA hyposensitivity phenotype, however, correlate with stress-tolerance phenotypes, it needs to be further proved.

Together, these results point to an important function of HAI-1 as a negative regulator of ABA signal transduction events. The identification of a negative regulator in ABA signaling based on a cDNA over-expression screen shows that this approach can be used to isolate mutants in genes that modulate complex signaling networks in plants [16, 18, 46].

The expression of HAI-1 is very low under normal growth condition, detected ubiquitously in various organs according to microarray analysis (AtGenExpress), expressed mainly in the stem. However, the expression of HAI-1 is stronger induced by biotic and abiotic stress factors. Our results demonstrated that GUS activity could be detected in pHAI-1::GUS transgenic plants subjected to ABA and wounding. In unstressed conditions, ABI1 transcripts were the most abundant of the group-A PP2Cs, whereas the HAI-1 expression level was comparatively lower. Conversely, under water stress conditions, HAI-1 was drastically up-regulated in comparison with ABI1 [13]. In this study, stronger GUS activity could be detected in pHAI-1::GUS in the background of WT plants either treated or untreated (e.g. ABA or wounding) conditions. The GUS activity could be detected at the distal part of the roots (Fig. 9d). After induction by stresses, the GUS activity was stronger induced in roots, stem and around wounded area such as leaf vein (Fig. 9e). Basal transcript levels of At5g59220 are lower than those reported for other clade A PP2Cs; however, its expression is highly induced by ABA or osmotic stress [13, 56]. The experiment results are consistent with the previous research.

Interaction of PP2CA with the plasma membrane transporters AKT2 and SLAC1 has been reported [6, 28] and interaction of PP2CA and At5g59220 with SnRK2 s was localized to both nucleus and cytosol [13]. We found At5g59220 localized in the nucleus through bombardment assay (Fig. 10).

Therefore, At5g59220 overexpression leads to reduce seed germination, as compared with untransformed plants under inhibitory concentrations of ABA or high-osmoticum media. Finally, super-induction of ABA- and stress-inducible genes in overexpression transgenic plants do not appear to be sufficient to induce drought avoidance. Biochemical analysis of HAI-1 gene are lack, need to further studies on its activity.
